# Obituary

**Published:** 1885-11

**Authors:** 


### OBITUARY.

---

DR. JOHN L. ATLEE died at his residence in Lancaster, Pennsylvania, on October 1st, at the advanced age of eighty-five years. Until about one year ago he enjoyed exceptional mental and physical vigor for his period of life. He was engaged in the active practice of his profession for over sixty years. He was an Ex-President of the American Medical Association. During his long and eventful life he filled many positions of responsibility with ability and scrupulous fidelity. He left one son surviving him, DR. WALTER L. ATLEE, of Philadelphia.

---

PROFESSOR RICHARD MCSHERRY, M. D., died at his residence in Baltimore, Maryland, on October 7th, 1885. In early life he had been an assistant-surgeon in the United States Army, from which he resigned in 1843. He then entered the United States Navy as an assistant-surgeon, and remained until 1851, when he resigned his commission. Subsequently he located in Baltimore, and, for many years, he has been known as one of the most prominent members of the medical profession of that city. He was sixty-eight years of age.

